# Assessing somatization in urologic chronic pelvic pain syndrome

**DOI:** 10.1186/s12894-019-0556-3

**Published:** 2019-12-10

**Authors:** C. S. North, B. A. Hong, H. H. Lai, D. H. Alpers

**Affiliations:** 1 The Altshuler Center for Education & Research at Metrocare Services, The University of Texas Southwestern Medical Center, 1250 Mockingbird Lane, Suite 330, Dallas, TX 75247-4914 USA; 2 Department of Psychiatry, The University of Texas Southwestern Medical Center, 5323 Harry Hines Blvd., Suite NE5.102, Dallas, TX 75390-9070 USA; 3 Department of Psychiatry, School of Medicine, Washington University School of Medicine, St. Louis, MO USA; 4 Departments of Surgery and Anesthesiology, Division of Urologic Surgery, Washington University School of Medicine, St. Louis, MO 63110 USA; 5 Department of Internal Medicine, Division of Gastroenterology, Washington University School of Medicine, St. Louis, MO 63110 USA

**Keywords:** Interstitial cystitis, Urological chronic pelvic pain syndrome, Chronic prostatitis, Somatization disorder, Symptom screening, Psychiatric diagnosis, Polysymptomatic, Polysyndromic, Somatoform, Psychoform

## Abstract

**Background:**

This study examined the prevalence of somatization disorder in Urological Chronic Pelvic Pain Syndrome (UCPPS) and the utility of two self-report symptom screening tools for assessment of somatization in patients with UCPPS.

**Methods:**

The study sample included 65 patients with UCPPS who enrolled in the Multidisciplinary Approach to the Study of Chronic Pelvic Pain (MAPP) Study at Washington University. Patients completed the PolySymptomatic PolySyndromic Questionnaire (PSPS-Q) (*n* = 64) and the Patient Health Questionnaire-15 Somatic Symptom Severity Scale (PHQ-15) (*n* = 50). Review of patient medical records found that only 47% (*n* = 30) contained sufficient documentation to assess Perley-Guze criteria for somatization disorder.

**Results:**

Few (only 6.5%) of the UCPPS sample met Perley-Guze criteria for definite somatization disorder. Perley-Guze somatization disorder was predicted by definite PSPS-Q somatization with at least 75% sensitivity and specificity. Perley-Guze somatization disorder was predicted by severe (> 15) PHQ-15 threshold that had > 90% sensitivity and specificity but was met by only 16% of patients. The moderate (> 10) PHQ-15 threshold had higher sensitivity (100%) but lower specificity (52%) and was met by 52% of the sample.

**Conclusions:**

The PHQ-15 is brief, but it measures symptoms constituting only one dimension of somatization. The PSPS-Q uniquely captures two conceptual dimensions inherent in the definition of somatization disorder, both number of symptoms and symptom distribution across multiple organ systems, with relevance for UCPPS as a syndrome that is not just a collection of urological symptoms but a broader syndrome with symptoms extending beyond the urological system.

## Introduction

Urological chronic pelvic pain syndrome (UCPPS), which includes interstitial cystitis/bladder pain syndrome (IC/BPS) and chronic prostatitis/chronic pelvic pain syndrome (CP/CPPS), is a symptom-based syndrome without objective testing or diagnostic biomarkers. UCPPS is diagnosed only through assessment of symptoms and symptom patterns reported by patients with this disorder. Bothersome symptoms characterizing UCPPS include not only the core urological symptoms of pelvic pain and urinary frequency/urgency but also an auxiliary halo of non-urologic pain (e.g., abdominal pain) and other symptoms extending beyond the urological system (e.g., nausea, dizziness, or palpitations) [3, 6, 16, 17].

To avoid unnecessary medical tests, procedures, and surgeries, it is important to sort out symptoms of somatoform disorders from medical illness, but this task has historically proven difficult for both psychiatrists and non-psychiatric physicians. Like many other functional disorders, UCPPS has many symptoms in common with somatization disorder, and patients diagnosed with UCPPS may include some patients who have somatoform disorders. In the current version of American diagnostic criteria for psychiatric disorders, the *Diagnostic and Statistical Manual of Mental Disorders*, 5th edition (*DSM-5*), [2] the previous diagnostic section on somatoform disorders was replaced with a section covering somatic symptom and related disorders; the former diagnosis of somatization disorder has been replaced by somatic symptom disorder, which does not require specified numbers of symptoms across organ systems or symptoms that are “medically unexplained.”

Some patients with UCPPS report not just symptoms confined to the definition of the syndrome but also multiple symptoms across multiple organ systems. One-fourth of patients with UCPPS have been observed to have this pattern of symptoms distributed widely across organ systems [16, 17]. This symptom presentation has been termed “polysymptomatic, polysyndromic”, [17, 21] and it is reminiscent of the classic presentation of somatization disorder. Somatization disorder has historically been well known to psychiatrists and other physicians as a chronic disorder defined by the presentation of multiple symptoms distributed across many organ systems. To represent somatization disorder, the symptoms had to be determined to have no medically explainable etiology. Like UCPPS, somatization disorder requires documentation of characteristic symptoms and symptom patterns reported by patients, because there are no objective tests for biomarkers to identify the disorder. For many years, these two symptom-based syndromes may have coexisted without recognition of the overlap between them.

The diagnosis of somatization disorder (under the name Briquet’s syndrome) has historically been made using criteria that were first established by Perley and Guze [23] and formally validated in 1972 [7]. The Perley-Guze criteria are recognized as the original, well-validated, and most comprehensive criteria for this diagnosis [7, 21, 23, 29]. The original Perley-Guze criteria continue to be accepted as valid for diagnosis of somatization disorder, regarded by some experts as superior to (and somewhat more stringent than) the most recently established criteria [1] for the disorder [21]. The Perley-Guze criteria remain meaningful today because psychiatrists and non-psychiatric physicians still need to understand the clinical significance of the symptoms their patients report. Although the Perley-Guze criteria have no relevance for making a *DSM-5* diagnosis of somatic symptom disorder, their utility for sorting out symptoms and symptom patterns is still important.

Various somatic symptom measures have been developed and used in different populations and settings for assessment of somatic symptoms and somatization syndromes. The PolySymptomatic PolySyndromic Questionnaire (PSPS-Q) developed by Lai et al. [17] has been used in UCPPS research to identify not only the large numbers of somatic symptoms reported by these patients but also their distribution across multiple organ systems that are characteristic of somatoform disorders. The PSPS-Q was conceptually derived from the Perley-Guze criteria. The Patient Health Questionnaire-15 Somatic Symptom Severity Scale (PHQ-15, 13] has also been used to assess somatization symptoms in patients with UCPPS.

The purpose of this study was to examine the prevalence of somatization disorder in UCPPS using the Perley-Guze criteria and to compare two self-report symptom screening tools—the PSPS-Q and the PHQ-15 to assist the evaluation of UCPPS. Its findings should be of interest to many psychiatrists, urologists, and primary care physicians who need to evaluate the medical symptoms in their patients.

## Methods

A sample of 65 patients with UCPPS was recruited from consecutive patients presenting to the Washington University urology clinic in the early years of the Multidisciplinary Approach to the Study of Chronic Pelvic Pain (MAPP) project at one site. Study inclusion criteria were 1) age > 18 years, 2) report of an unpleasant sensation of pain, pressure, or discomfort perceived by the patient to involve the bladder and/or pelvic region and associated with lower urinary tract symptoms, e.g., urgency, for most of the time during the most recent 3 months, and 3) symptoms were not explained by another identifiable urological cause. Exclusion criteria were active urinary tract infection, cancer, urethral stricture, neurological disease, fistula, radiation cystitis, cyclophosphamide cystitis, urinary tuberculosis, or open bladder surgery. These inclusion and exclusion criteria were adapted from the MAPP Research Network working definition of UCPPS [18]. Approval for this study was obtained from the Institutional Review Board of Washington University, and written informed consent was provided by all patients at the time of their enrollment into the study.

Data for this study were collected between March 2010 and October 2012. As part of the MAPP study, participants provided information on demographics, symptom duration; ratings of their pain; frequency and urgency on 0–10 numeric ratings scales; Interstitial Cystitis Symptom and Problem Indexes [22]; the Genitourinary Pain Index [4]; the Complex Medical Symptoms Inventory to assess symptoms of irritable bowel syndrome (IBS) or fibromyalgia [28]; and a whole body map to characterize the location and distribution of pain in the past week [15]. A full list of data collected from the MAPP Epidemiology and Phenotyping Study has previously been described [18].

The Washington University electronic outpatient medical records of the patients in this sample were reviewed by an expert psychiatric diagnostician (C.S.N.), a urologist (H.H.L.), and an experienced medical/clinical psychologist (B.A.H.) to formally assess Perley-Guze criteria for somatization disorder. These 3 clinicians from different areas of expertise provided multidisciplinary consensus through interactive discussion of each patient’s medical history in the clinical records. A definite diagnosis requires > 25 symptoms in > 9 symptom categories, but probable diagnosis (requiring > 20 symptoms in > 9 symptom categories) has been utilized historically to include histories strongly suggestive of somatization disorder but not quite meeting sufficient numbers of symptoms [7, 23]. The probable diagnosis option was formalized for use in suboptimal circumstances affording insufficient data for complete assessment—such as the current study in which investigators were unable to obtain data through collection of clinical information directly from patients over long periods of time.

At the baseline clinic visit, demographic information was recorded and the patients completed the PSPS-Q and the PHQ-15. The PSPS-Q is a self-report symptom questionnaire assessing 59 somatic symptoms across 10 symptom categories, derived from the 59-item yes/no Perley-Guze symptom checklist and also requiring > 25 symptoms distributed among > 9 of 10 possible symptom categories [20]. Only symptoms considered by the patient to represent “a lot of trouble” are counted as Perley-Guze symptoms. The PSPS-Q, unlike the original Perley-Guze criteria historically used in clinical practice and research, does not exclude medically unexplained symptoms. The symptom count/symptom group scoring algorithm used for determining the original Perley-Guze criteria was applied for scoring the PSPS-Q data. Because the 4 menstrual symptoms comprising 1 of the 10 Perley-Guze symptom groups do not apply to men, the PSPS-Q scoring threshold for men was reduced to > 21 symptoms in > 8 categories for definite somatization and to > 16 symptoms in > 8 categories for probable somatization.

The PHQ-15 is a self-report symptom questionnaire derived from the Patient Health Questionnaire that consists of 15 items scored from 0 (“not bothered at all”) to 2 (“bothered a lot”), with scores of 5, 10, and 15 respectively representing mild, moderate, and severe somatization. The PHQ-15 has demonstrated validity and reliability and is widely used for screening and monitoring somatization and somatic symptom severity in clinical practice and research [12–14, 25]. The moderate PHQ-15 threshold score of > 10 has been demonstrated to detect patients with medically unexplained physical symptoms with 30% sensitivity and 93% specificity, and with positive predictive and negative predictive values (PPV and NPV) of .40 and .89 respectively [5].

The PSPS-Q was completed by 64 patients and the PHQ-15 was completed by 50 of these patients. Probable and definite self-report PSPS-Q somatization were not associated with non-completion of the PHQ-15.

### Statistical analysis

Data analysis for this study was conducted using SAS version 9.4 (SAS Institute, Cary, NC). Descriptive findings are represented as counts with proportions and means with standard deviations (SD). Comparisons of numerical variables with a dichotomous variable for sex used Wilcoxon two-sample rank sum tests (PROC NPAR1WAY WILCOXON in SAS) using t approximations with a continuity correction of 0.5. Dichotomous variables generated by different forms of assessment were compared through tests of sensitivity, specificity, PPV, and NPV, as well as with chi-square tests.

## Results

The 64 patients with PSPS-Q data and medical record review was 48% male, 86% non-Hispanic white, 6% Hispanic, and 8% other race/ethnicity. The sample had a mean (SD) age of 46.8 (16.1) years. The mean (SD) duration of UCPPS symptoms was 10.2 (12.0) years. IBS was present in 30%, fibromyalgia in 8%, and chronic fatigue syndrome in 13%. Mean (SD) ratings on a scale of 1–10 were 4.9 (2.4) for pain, 4.8 (2.8) for frequency, and 5.0 (2.6) for urgency. The mean (SD) genitourinary pain index total score was 24.2 (9.6). The mean (SD) Interstitial Cystitis Symptom and Problem Indexes were 9.5 (5.2) and 8.2 (4.6) respectively. The mean (SD) number of body sites outside the pelvis that had pain was 3.8 (3.6) (of a maximum of 42 sites). Of the 34 female patients (52% of the sample), all had the clinical diagnosis of interstitial cystitis/bladder pain syndrome (IC/BPS). Of the 31 male patients (48% of the sample), 28 had the clinical diagnosis of chronic prostatitis/chronic pelvic pain syndrome (CP/CPPS), and 3 had the dual diagnoses of IC/BPS and CP/CPPS.

The medical records of more than one-half of the patients (53%, 34/64) were found to contain insufficient documentation (largely because of a lack of medical records by primary care providers and clinicians of other disciplines) to allow the identification of patterns of multiple somatoform symptoms across multiple organ systems; thus, the medical records of only 30 patients could be used to assess Perley-Guze criteria for somatization disorder.

Somatization disorder was uncommon in this UCPPS sample: only 2 patients (6.5%) met definite Perley-Guze criteria based on medical record review. Two additional patients (6.5%) met probable Perley-Guze criteria. All 4 of these patients were female.

The mean (SD) number of somatic symptoms on the self-report PSPS-Q was 14.9 (11.2) and the mean (SD) number of symptom groups was 6.4 (3.0). Men and women did not differ on numbers of PSPS-Q symptoms or symptom groups. One-third (33%, 21/64) of the sample met the threshold for at least probable PSPS-Q somatization, including 17/64 (27%) with definite PSPS-Q somatization. Insufficient medical record documentation was not associated with number of symptoms or positive symptom groups reported on the PSPS-Q.

Among the 30 patients with sufficient medical records, 11 (37%) met the probable PSPS-Q somatization threshold, including 8 (27%) who met the definite threshold. Medical record review found that 38% of the 8 patients meeting the definite PSPS-Q threshold met Perley-Guze criteria for somatization disorder (2 probable, 1 definite). The fourth patient meeting (definite) Perley-Guze criteria for somatization disorder by medical record review was not forthcoming on the PSPS-Q, reporting only 2 symptoms in 1 symptom group.

Table 1 presents data for prediction of definite and probable Perley-Guze somatization disorder criteria by medical record review from definite and probable PSPS-Q somatization. The definite PSPS-Q threshold was significantly associated by chi-square test with probable Perley-Guze somatization disorder, which it detected with sensitivity and specificity levels of at least 75% and positive predictive value of 38%.

**Table 1:** Definite and probable PSPS prediction of definite and probable Perley-Guze somatization disorder by medical record review (*n* = 30)

	Sensitivity	Specificity	PPV	NPV	p
Predicting definite Perley-Guze somatization disorder by medical record review (7% of the sample)					
Definite PSPS (27% positive)	50%	75%	13%	95%	.469
Probable PSPS (37% positive)	50%	64%	9%	95%	1.000
Predicting probable Perley-Guze somatization disorder by medical record review (13% of the sample)					
Definite PSPS (27% positive)	75%	81%	38%	95%	.048
Probable PSPS (37% positive)	75%	69%	27%	95%	.126

*PPV* positive predictive value, *NPV* negative predictive value, *PSPS* polysymptomatic, polysyndromic, *PSPS-Q* PSPS Questionnaire

The mean (SD) PHQ-15 score was 8.7 (4.4), representing a mild-to-moderate level of somatization; 42% (21/50) scored at or above the moderate threshold of 10 and 10% (5/50) scored at or above the severe threshold of 15. PHQ-15 scores were higher for women than for men (mean = 10.3, mean = 4.7 vs. mean = 6.7, SD = 2.9; z = 2.86, *p* = .004. Insufficient medical record documentation was not associated with PHQ-15 scores.

Table 2 presents results for prediction of definite Perley-Guze somatization disorder by medical record review from PHQ-15 thresholds representing mild, moderate, and severe somatization. (Because the 2 patients with probable but not definite somatization disorder by medical record review did not complete the PHQ-15, prediction of probable somatization disorder by the PHQ-15 was not possible.) The moderate PHQ-15 threshold > 10 identified 52% of the sample and predicted definite Perley-Guze somatization disorder with 100% sensitivity but only 52% specificity and a PPV of only 15%, not meeting statistical significance by chi-square test. A severe PHQ-15 score > 15, however, identified just 16% of the sample, and this threshold significantly predicted definite Perley-Guze somatization disorder by chi-square test with at least 90% for both sensitivity and specificity, and with a PPV of 50%. Half (2 of the 4) of the patients with a severe PHQ-15 score > 15 had definite Perley-Guze somatization disorder by medical record review.

**Table 2:** PHQ-15 prediction of definite Perley-Guze somatization disorder by medical record review (*n* = 25)

	Sensitivity	Specificity	PPV	NPV	p
PHQ-15 threshold					
5 (92% positive)	100%	9%	9%	100%	.664
10 (52% positive)	100%	52%	15%	100%	.480
15 (16% positive)	100%	91%	50%	100%	.020

*PPV* positive predictive value, *NPV* negative predictive value

## Discussion

This study examined and compared data from a 59-item self-report symptom questionnaire (PSPS-Q) and a 15-item self-report screening questionnaire (PHQ-15) with medical record review to assess Perley-Guze criteria for somatization disorder in a sample of patients with UCPPS. Medical record review by 3 experts confirmed that 6.5% of the patients with UCPPS had definite and another 6.5% had probable or definite somatization disorder. Thus, it was uncommon for UCPPS patients to meet definite Perley-Guze criteria for somatization disorder (only 6.5%). This frequency is similar to the 1–6% point prevalence of somatization disorder in primary care patients reported by a recent systematic review that identified, from a total of 992 publications, 32 studies from 24 countries involving > 70,00 patients [9]. Self-reported PSPS-Q somatization (both probable and definite thresholds) had higher sensitivity and specificity for prediction of probable than for definite Perley-Guze somatization disorder verified by medical record review.

Perley-Guze somatization disorder verified by medical record review was predicted by both self-reported questionnaires. Probable Perley-Guze somatization disorder was predicted by PSPS-Q definite somatization with at least 75% sensitivity and specificity, and by moderate (threshold > 10) PHQ-15 scores with higher sensitivity (100%) but lower specificity (52%). These two comparisons were not completely comparable, however, because the PSPS-Q comparison included patients with both definite (*n* = 2) and probable (*n* = 2) Perley-Guze somatization disorder but the PHQ-15 comparison could not include the 2 probable Perley-Guze somatization disorder cases who did not complete the PHQ-15. Neither definite nor probable PSPS-Q somatization were significantly associated with moderate or higher PHQ-15 scores. The prediction of definite Perley-Guze somatization disorder by the PHQ-15 was statistically significant using a higher PHQ-15 threshold (> 15, representing severe somatization), with > 90% sensitivity as well as specificity. Using the severe PHQ-15 threshold also substantially reduced the patient pool under consideration to 16% of all patients versus 52% selected by the moderate PHQ-15 threshold. In summary, the PSPS-Q had greater sensitivity but lower specificity than the moderate PHQ-15 threshold that has been previously reported for identifying somatization disorder, and a higher (severe) PHQ-15 somatization threshold performed better than the PHQ-15 moderate somatization threshold for prediction of Perley-Guze somatization disorder.

A limitation to attempting to assess somatization disorder criteria by any self-report questionnaire is that patients cannot be expected to determine whether their own symptoms are medically explained or unexplained. For this reason, self-report symptom questionnaires cannot be used to diagnose somatization disorder [17, 21]. Two patient reporting behaviors further limit self-report questionnaires: 1) patients with somatization disorder do not report all their symptoms at once, focusing their medical complaints on or even limiting them to the organ system of the specialist, which necessitates collection of symptoms across different visits and to different medical specialists [19, 20]; 2) these patients may provide medically plausible but incorrect explanations for their symptoms and may even attribute their symptoms to medical disorders they do not have [11, 19].

Limitations of this study include the small sample selected in a treatment setting and investigated at a single site from a specialty service at an academic institution that may not be representative of other samples and settings. Verification of diagnoses of somatization disorder through medical record review was not possible for more than half of the patients in this study because the available patient records were limited to one or very few visits to a limited set of providers, likely resulting in substantial underestimation. The small sample size coupled with low numbers of patients meeting Perley-Guze criteria for somatization disorder by medical record review resulted in low power to detect subgroup differences; regardless, analyses in this study suggested patterns of association of symptom measures with the diagnosis of somatization disorder which deserve further study with larger samples in settings with more complete medical records.

Not only has the traditional means of assessing diagnostic criteria through medical record review been lost with the evolution to current medical record systems, but the diagnosis of somatization disorder itself has been lost from established diagnostic criteria in *DSM-5*. This loss is still controversial, and somatization disorder is recognized as one of the few validated psychiatric disorders, consistently included in previous versions of the manual (and remains part of the International Classification of Diseases system). This diagnosis was replaced in *DSM-5* with somatic symptom disorder, an unvalidated syndrome that is overly broad and inappropriately inclusive of patients with nonpathological distress attributable to medical illness [8, 10, 24, 26, 27].

Identifying patients with polysymptomatic, polysyndromic symptom-reporting patterns characteristic of somatization disorder in patient populations with UCPPS is of clinical importance because their medical outcomes are distinctly worse than those of other patients. These patients may require different clinical interventions, to avoid unnecessary medical tests, procedures, and surgeries. The prevalence of somatization disorder in UCPPS is unknown despite attempts to estimate it using various symptom measures and methods.

Without the time-honored tools historically used to identify somatization syndromes through detailed medical records and longitudinal assessment of patients’ evolving symptoms, other means of identifying patterns of somatization are needed. The PSPS-Q or the PHQ-15 might be useful for identifying polysymptomatic, polysyndromic presentations by completing a screening tool in the pre-appointment waiting area to systematically address symptoms outside the urinary system that are not the focus of the brief clinical history gathered by urologists. This information might prompt urologists to collect additional history to identify patterns of multiple symptoms distributed across multiple organ systems that is characteristic of somatization disorder.

An advantage of the PHQ-15 over the PSPS-Q is that it is less burdensome to complete, with only 15 items compared to 59 in the PSPS-Q. However, the PSPS-Q uniquely captures two conceptual dimensions inherent in the definition of somatization disorder rather than one: it not only counts the number of symptoms, but it also assesses the extent of symptom distribution across multiple organ systems that is integral to the conceptualization and diagnosis of somatization disorder. Consideration not just of number of symptoms but also their distribution across organ systems has relevance for the appreciation of UCPPS as a syndrome that is not just a collection of urological symptoms but a broader syndrome with symptoms extending beyond the urological system.

Although the self-report PSPS-Q does not exclude medically unexplained symptoms, the Perley-Guze symptom algorithm has been found to retain its validity even without this exclusion [29]. Brief symptom measures with as few as 15 items cannot be expected to assess both the volume of symptoms and their distribution throughout the organ systems that is provided with the 59 symptom items of the PSPS-Q.

## Conclusions

The PHQ-15 is brief, but it measures symptoms constituting only one dimension of somatization. The PSPS-Q uniquely captures two conceptual dimensions inherent in the definition of somatization disorder, both number of symptoms and symptom distribution across multiple organ systems, with relevance for UCPPS as a syndrome that is not just a collection of urological symptoms but a broader syndrome with symptoms extending beyond the urological system. Few UCPPS patients (only 6.5%) met definite Perley-Guze criteria for somatization disorder by medical record review, but the PSPS-Q identified somatization in 27% of the sample and the PHQ-15 severe threshold identified somatization in 16%.

## Data Availability

The dataset generated for the current study are not publicly available because of ownership by the MAPP Study, but the data can be provided by the authors on reasonable request to the corresponding author.

## Abbreviations

CP/CPPS: Chronic prostatitis/chronic pelvic pain syndrome
DSM-5: Diagnostic and Statistical Manual of Mental Disorders, 5th edition
IBS: Irritable bowel syndrome
IC/BPS: Interstitial cystitis/bladder pain syndrome
MAPP: Multidisciplinary Approach to the Study of Chronic Pelvic Pain
NPV: Negative predictive value
PHQ-15: Patient Health Questionnaire-15 Somatic Symptom Severity Scale
PPV: Positive predictive value
PSPS-Q: PolySymptomatic PolySyndromic Questionnaire
SD: Standard deviation
UCPPS: Urological Chronic Pelvic Pain Syndrome
